# DRED: A Comprehensive Database of Genes Related to Repeat Expansion Diseases

**DOI:** 10.1093/gpbjnl/qzae068

**Published:** 2024-09-30

**Authors:** Qingqing Shi, Min Dai, Yingke Ma, Jun Liu, Xiuying Liu, Xiu-Jie Wang

**Affiliations:** Key Laboratory of Genetic Network Biology, Institute of Genetics and Developmental Biology, Chinese Academy of Sciences, Beijing 100101, China; Key Laboratory of Genetic Network Biology, Institute of Genetics and Developmental Biology, Chinese Academy of Sciences, Beijing 100101, China; University of Chinese Academy of Sciences, Beijing 100049, China; Key Laboratory of Genetic Network Biology, Institute of Genetics and Developmental Biology, Chinese Academy of Sciences, Beijing 100101, China; Key Laboratory of Genetic Network Biology, Institute of Genetics and Developmental Biology, Chinese Academy of Sciences, Beijing 100101, China; Key Laboratory of Genetic Network Biology, Institute of Genetics and Developmental Biology, Chinese Academy of Sciences, Beijing 100101, China; Key Laboratory of Genetic Network Biology, Institute of Genetics and Developmental Biology, Chinese Academy of Sciences, Beijing 100101, China; University of Chinese Academy of Sciences, Beijing 100049, China

**Keywords:** Repeat expansion, Disease, Short tandem repeat, Genetic variation, Trinucleotide repeat

## Abstract

Expansion of tandem repeats in genes often causes severe diseases, such as fragile X syndrome, Huntington’s disease, and spinocerebellar ataxia. However, information on genes associated with repeat expansion diseases is scattered throughout the literature, systematic prediction of potential genes that may cause diseases via repeat expansion is also lacking. Here, we develop DRED, a Database of genes related to Repeat Expansion Diseases, as a manually-curated database that covers all known 61 genes related to repeat expansion diseases reported in PubMed and OMIM, along with the detailed repeat information for each gene. DRED also includes 516 genes with the potential to cause diseases via repeat expansion, which were predicted based on their repeat composition, genetic variations, genomic features, and disease associations. Various types of information on repeat expansion diseases and their corresponding genes/repeats are presented in DRED, together with links to external resources, such as NCBI and ClinVar. DRED provides user-friendly interfaces with comprehensive functions, and can serve as a central data resource for basic research and repeat expansion disease-related medical diagnosis. DRED is freely accessible at http://omicslab.genetics.ac.cn/dred, and will be frequently updated to include newly reported genes related to repeat expansion diseases.

## Introduction

Repeated sequences, also known as repetitive elements, comprise more than 50% of the human genome, among which millions are short tandem repeats (STRs) with typical repeat length of 2–6 bp [1–3]. Although the majority of STRs are located in intergenic noncoding regions, many human coding genes also harbor STRs in exons or introns [4,5]. Copy number variation of repeat units is commonly seen among STRs, which may be caused by polymerase slippage during the DNA replication, repair, and recombination processes [6–8]. Abnormal expansion of STRs can lead to gene dysfunction at the RNA or protein level, and result in more than 40 severe inherited diseases [2,3,9–12]. Notably, RNAs with expanded repeats can independently promote phase separation and gelation, forming RNA foci in the nuclei [13–15]. Most repeat expansion-related disorders are neurological, neuromuscular, or neurodegenerative diseases, such as the (CGG)*_n_* repeats in fragile X syndrome, (CAG)*_n_* repeats in Huntington’s disease, and (GAA)*_n_* repeats in Friedreich’s ataxia [16–20]. For these diseases, the expansion of STRs is usually non-toxic when the copy numbers of STRs are below certain threshold; however, along cell division, the expansion of STRs can accumulate and become pathogenic, and result in severe symptoms. The repeat expansion diseases usually have earlier onset time in descendent generations, such phenomenon is known as genetic anticipation and is a hallmark of repeat expansion diseases [18,20,21].

The majority of known disease-causing repeats are trinucleotide tandem repeats, with CAG (encoding polyglutamine) and GCG (encoding polyalanine) being the most prevalent STRs within protein-coding regions [22,23]. Multiple factors at the *cis*-regulation level could promote the expansion of STRs, including repeats located within or adjacent to CpG islands [24], mutations in adjacent CCCTC-binding factor (CTCF) binding sites [25], and the presence of nearby *Alu* elements [26,27] or topological associating domain (TAD) boundaries [28].

Here, we present DRED as the first database of genes related to repeat expansion diseases. DRED not only encompasses comprehensive information on known causal genes for repeat expansion diseases, but also provides a list of predicted genes with the potential to cause diseases via repeat expansion, therefore may help researchers to identify unknown repeat expansion diseases and novel disease-causing genes.

## Database contents and construction

### Database contents

**Figure 1:**
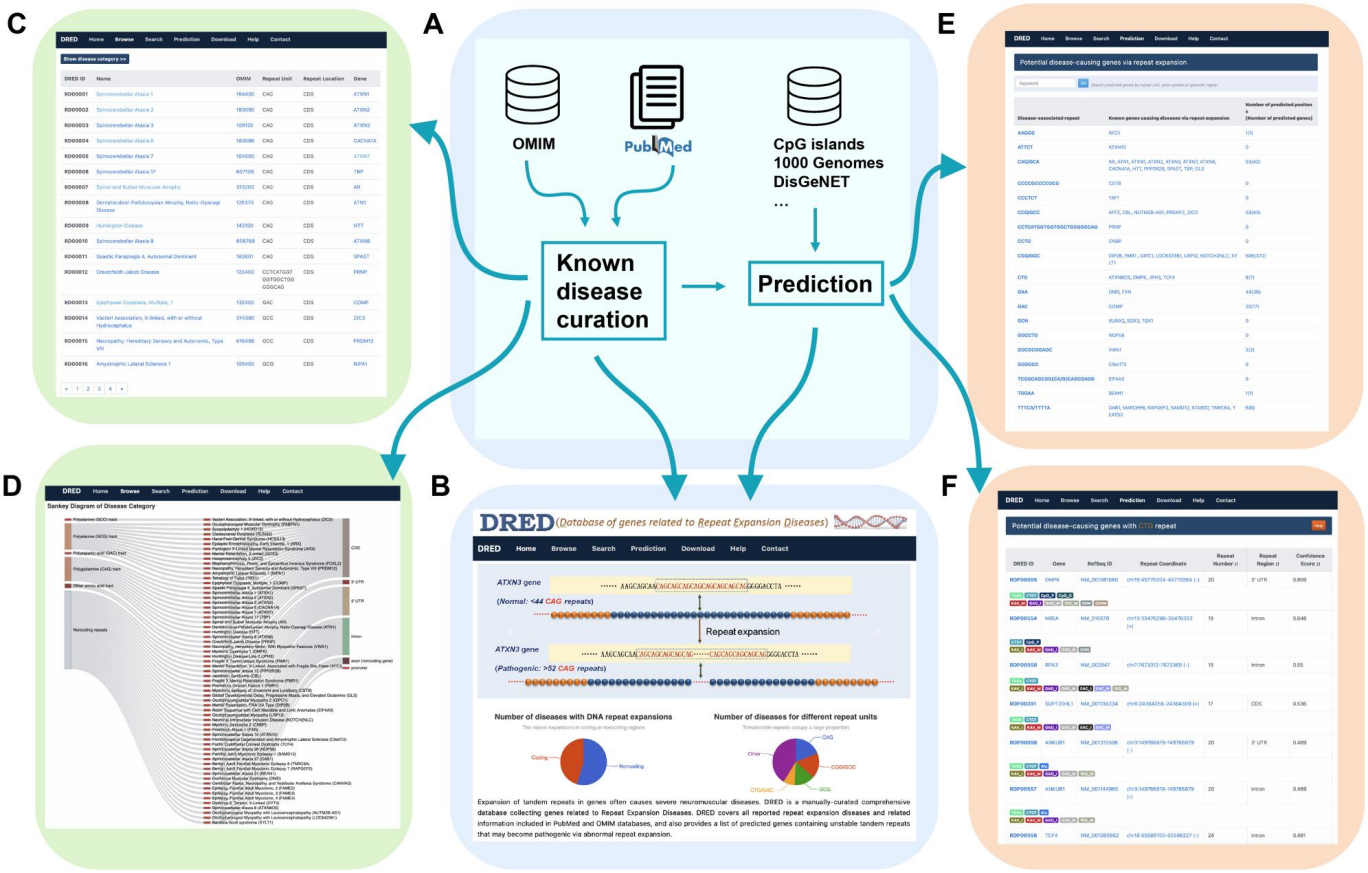
Scheme and functional illustration of DRED **A**. Design scheme of DRED. **B**. Function overview of DRED. **C**. List of known repeat expansion diseases under the Browse function. **D**. Interactive Sankey diagram showing the categories of known repeat expansion diseases. **E**. Overview of the predicted disease-causing genes. **F**. Availability of external information for the predicted disease-causing genes. DRED, Database of genes related to Repeat Expansion Diseases; CDS, coding sequence; UTR, untranslated region.

DRED contains all reported 61 genes related to 62 known repeat expansion diseases or disease subtypes collected in the PubMed or OMIM databases (**Figure 1**A and B). For each disease or disease subtype, its phenotype and general information, pathogenic gene, pathogenic repeat, repeat conservation status, pathogeny, and related references are included. Links to external data resources, such as Kyoto Encyclopedia of Genes and Genomes (KEGG) [29], Gene Ontology (GO) [30,31], and ClinVar [32], are also provided. The expandable STRs of these 61 genes can be classified into 22 types, with CAG, CGG, GCG, and TTTCA/TTTTA as the most commonly observed expandable STR types (**Table 1**). The distributions of known disease-causing STRs are comparable across 5′ untranslated regions (UTRs), introns, and coding sequences (CDSs), but are under-presented in exons or 3′ UTRs of noncoding genes (Table 1). Among the known repeat expansion diseases, only spinocerebellar ataxia type 8 and oculopharyngeal myopathy with leukoencephalopathy (OPML) are caused by STRs within noncoding genes, namely *ATXN8OS* [33] and *LOC642361/NUTM2B-AS1* [34], respectively. It is worth noting that a total of 12 subtypes of spinocerebellar ataxias are related to repeat expansion, among which 7 are caused by abnormal expansion of CAG repeats encoding polyglutamine tracts in different genes [35,36].

**Table 1:** Summary of the known repeat expansion disease-causing genes collected in DRED

Repeat unit	Location	Gene
AAGGG ATTCT	Intron Intron	*RFC1* *ATXN10*
CAG	5′ UTR, CDS	*AR* ^[1]^, *ATN1*^[1]^, *ATXN1*^[1]^, *ATXN2*^[1]^, *ATXN3*^[1]^, *ATXN7*^[1]^, *ATXN8*^[1]^, *CACNA1A*^[1]^, *HTT*^[1]^, *PPP2R2B*^[2]^, *SPAST*^[1]^, *TBP*^[1]^
CCCCGCCCCGCG	5′ UTR	*CSTB*
CCCTCT	Intron	*TAF1*
CCG	5′ UTR, exon (noncoding gene)	*AFF2* ^[2]^, *CBL*^[2]^, *NUTM2B-AS1*^[3]^
CCTCATGGTGGTGGCTGGGGGCAG	CDS	*PRNP*
CCTG	Intron	*CNBP*
CGG	Promoter, 5′ UTR, exon (noncoding gene)	*DIP2B* ^[2]^, *FMR1*^[2]^, *GIPC1*^[2]^, *LOC642361*^[3]^, *LRP12*^[2]^, *NOTCH2NLC*^[2]^, *XYLT1*^[4]^
CTG	Exon (noncoding gene), intron, 3′ UTR	*ATXN8OS* ^[3]^, *DMPK*^[5]^, *JPH3*^[5]^, *TCF4*^[6]^
GAA	Intron	*DMD*, *FXN*
GAC	CDS	*COMP*
GCA	5′ UTR	*GLS*
GCC	CDS	*PRDM12*, *ZIC3*
GCG	CDS	*ARX*, *FOXL2*, *HOXA13*, *HOXD13*, *NIPA1*, *PABPN1*, *PHOX2B*, *ZIC2*
GCN	CDS	*RUNX2*, *SOX3*, *TBX1*
GGCCTG	Intron	*NOP56*
GGCGCGGAGC	CDS	*VWA1*
GGGGCC	Intron	*C9orf72*
TCGGCAGCGG(CA/G)CAGCGAGG	5′ UTR	*EIF4A3*
TGGAA	5′ UTR	*BEAN1*
TTTCA/TTTTA	Intron	*DAB1*, *MARCHF6*, *RAPGEF2*, *SAMD12*, *STARD7*, *TNRC6A*, *YEATS2*

*Note*: [1], [2], [3], [4], [5], and [6] indicate that the repeat units are located in the CDS, 5′ UTR, exon (noncoding gene), promoter, 3′ UTR, and intron regions of genes, respectively. CDS, coding sequence; UTR, untranslated region.

To search for additional genes with the potential to induce diseases by repeat expansion, we collected sequence features known to contribute to the expansion of repeat sequences, and used an unsupervised machine learning algorithm to predict genes with the potential to induce diseases via repeat expansion. The features used for gene selection include: the presence of known disease-causing STRs, co-localization with *Alu* elements, CpG islands, CTCF binding sites, TAD boundaries, and sequence variations among populations and reported disease associations. A total of 516 candidate genes that may cause diseases via STR expansion were identified. These genes were classified by repeat types and included in the prediction section of DRED. For the predicted disease-causing STRs, DRED provides the information on putative expandable STRs, *cis*-elements adjacent to STRs, phylogenetic conservation of STRs, variations of STRs among populations, and STR-associated diseases. Links to the corresponding NCBI gene webpage, expression information [37], GO annotation [38], and the UCSC Genome Browser [39] are also provided.

### Web interface and usage

DRED provides user-friendly web interfaces with comprehensive functions as described below.

#### Browse

The browse function allows users to explore the comprehensive information of all known repeat expansion diseases (Figure 1C). The 62 known repeat expansion diseases are grouped by the features of their causal STRs, namely “Polyalanine (GCC) track”, “Polyalanine (GCG) track”, “Polyaspartic-acid (GAC) track”, “Polyglutamine (CAG) track”, “Other amino acid track”, and “Noncoding repeats” (Figure 1D). For each disease listed in the Browse page, detailed description on disease phenotype, disease-causing genes, pathogenic repeat unit, repeat length, related references, and other information can be obtained by corresponding links.

#### Search

The search function features a user-friendly interface that allows users to find specific information related to a repeat expansion disease. The search engine supports free-text queries, including disease names, gene symbols, repeat units, OMIM identifiers (IDs), chromosome numbers, or any keyword related to a disease. For example, entering the word “ataxia” will retrieve 18 entries with “ataxia” in the disease names or alternative disease names. An interactive 3D word cloud is provided in the search page to inform users the known repeat expansion diseases and their related genes in the database. Users can also pull up the detailed descriptions for each disease or gene by clicking on any term within the word cloud.

#### Prediction

**Figure 2:**
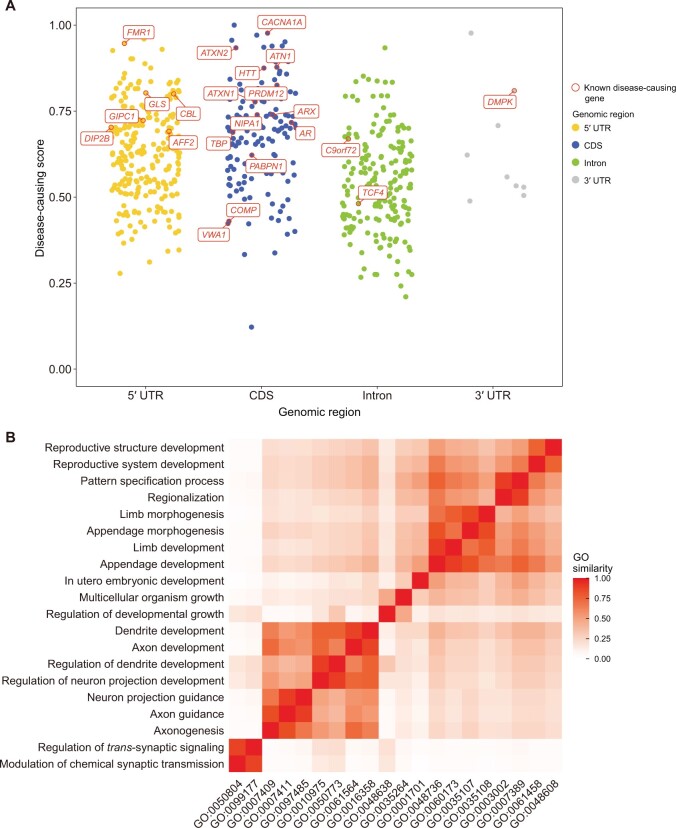
Characterization of predicted disease-causing genes **A**. Genomic distributions and prediction scores of the predicted disease-causing genes and the known ones. Known disease-causing genes are displayed in red boxes. **B**. GO enrichment analysis for the 516 predicted disease-causing genes. Shown are the top 20 enriched Biological Process terms. GO, Gene Ontology.

The prediction function provides a comprehensive list of genes with the potential to cause diseases via repeat expansion. A total of 516 genes (477 protein-coding genes and 39 noncoding genes) containing repeats belonging to 14 repeat units are included (Figure 1E). Users can retrieve all predicted genes with any repeat unit by clicking on either the repeat unit link or the corresponding gene count. For each gene, the detailed description, known repeat variations, and links to several external databases, are available via the link under DRED ID (Figure 1F). The prediction score and co-localization information of each gene with various *cis*-elements are also included. The genomic distribution features and predicted disease-causing scores of the predicted genes are similar to those of the known causal genes for repeat expansion diseases (**Figure 2**A). GO analysis using clusterProfiler [40] and GOSemSim [41] reveals an enrichment of terms related to neural system and limb development among the 516 potential disease-causing genes (Figure 2B), which is in concert with the neurological or neuromuscular related functions of most known repeat expansion diseases.

#### Download

All data collected in DRED are available for local manipulation through the download function. Information on known repeat expansion diseases and predicted disease-causing genes is provided in separate downloadable files.

## Conclusion

Abnormal expansion of STRs, mainly within protein-coding genes, is the causal factor for many neurological, neuromuscular, and neurodegenerative diseases. As these diseases are inheritable and have the genetic anticipation feature across generations, early diagnosis of risky repeat carriers may help to prevent or delay the onset of the diseases, especially during the era of precision medicine. In addition, the pathogenic mechanisms of most repeat expansion diseases remain elusive, effective prevention and treatment methods are in urgent demands. Although all known disease-causing repeat expansion elements are STRs, most STRs do not have expansions or give rise to repeat expansion diseases. The current available repeat-related databases only focus on general repeat sequences in genomes, which lack comprehensive information for human repeat expansion diseases [42–45]. To meet the needs from basic research and clinical diagnosis, we developed DRED, an integrative and user-friendly database for genes related to repeat expansion diseases. DRED not only contains comprehensive information on all known causal genes for repeat expansion diseases, but also provides a list of genes with the potential to cause diseases via abnormal repeat expansion. The candidate gene list may serve as a valuable resource for researchers and clinicians to identify new repeat expansion diseases or disease-causing genes, as well as to decipher their underlying molecular mechanisms. Continuously updated with new data every six months, DRED aims to be the premier resource for study, diagnosis, and treatment of repeat expansion diseases.

## Materials and methods

### Data collection and preprocessing

#### Repeat expansion disease collection from the literature

All known repeat expansion diseases were collected from the PubMed literature [46] and OMIM [47] databases using “repeat expansion”, “trinucleotide repeat expansion”, “triplet repeat expansion”, “repeat expansion disease”, and “repeat expansion disorder” as the query words (Figure 1A). In total, 6460 publications and 14,888 disease entries (retrieved on May 26, 2023) were manually curated to remove irrelevant information. A total of 62 repeat expansion diseases with supports from PubMed publications and/or OMIM records were retained in DRED.

#### Human genetic variations

Human genetic variants in different populations of the 1000 Genomes Project [48], the Known VARiants database (Kaviar) [49], the NHLBI GO Exome Sequencing Project (ESP) [50], the sequence variation and human phenotype database (ClinVar), the Exome Aggregation Consortium (ExAC) [51,52], and the Genome Aggregation Database (gnomAD) [53] (Table S1) were collected to examine the alterations of candidate STRs among individuals, and used as a criterion for candidate disease-causing gene prediction. Picard’s LiftoverVcf (http://broadinstitute.github.io/picard/) was used to convert Variant Call Format (VCF) files from the reference human genome build GRCh37 to GRCh38.

#### *Alu* elements, CpG islands, CTCF sites, and TAD boundaries

The genomic coordinates of *Alu* elements and CpG islands were extracted according to the human GRCh38 genome assembly presented by the UCSC Table Browser (http://genome.ucsc.edu/cgi-bin/hgTables). CTCF binding peaks were obtained from 10 CTCF ChIP-seq experiments in the ENCODE project using different human tissues/cell types (Table S2) [54]. Preprocessed TAD coordinates in 40 different human tissues/cell types were downloaded from 3D Genome Browser [55]; TAD boundaries of 200 kb (±100 kb centered on the boundary sites) in size were extracted using an in-house built script.

### Prediction of causal genes for repeat expansion diseases

To predict other genes with STRs that may be capable of causing diseases via repeat expansion, we firstly extracted the reported genomic and genetic features that could contribute to repeat expansion, including: (1) the presence of nearby *cis*-elements, such as *Alu* elements, CpG islands, CTCF binding sites, and TAD boundaries; (2) variation of STR copy numbers among populations, as evaluated using the 1000 Genomes, Kaviar, ESP, ClinVar, ExAC, and gnomAD databases; (3) the implication of genes in diseases according to information in the OMIM or DisGeNET [56] databases. Details of these features are listed in Table S3. The repeat-containing genes were then selected from the GRCh38 human genome with the following criteria: (1) the genomic sequences of a gene should contain STRs with copy numbers no less than the median value of the STR copy number range associated with normal phenotypes; (2) STRs within a gene should have at least two copies of expansion in one or more records in the 1000 Genomes, Kaviar, ESP, ClinVar, ExAC, and gnomAD databases. In total, 567 STR sites from 516 genes were keep as the final prediction results in DRED.

In order to further prioritize the predicted disease-causing genes, we used principal component analysis (PCA) to identify genomic features enriched among these disease-associated genes using the prcomp() function in R. In the input matrix for PCA, each row is a gene and each column is a feature, and the first principal component (PC1) captured the major variations of the input matrix (62.9%). Next, we performed a min-max normalization for genes’ coordinates on PC1 and assigned the normalized value as the disease-causing score for each gene. The corresponding weights of the 19 different features on PC1 are listed in Table S3, and the top 3 weighted features were with reported STR expansion in gnomAD, the presence of CpG islands in proximity region, and overlapping of CpG islands with the repeat tracks.

### Database and web interface implementation

DRED runs on an apache web server and is implemented in PHP 5.6.31 (http://www.php.net). The server-side PHP scripts deal with SQL query for keywords submitted by users and then execute through MySQL 5.7.16 (https://www.mysql.com), and return query result via interactive web interfaces written in bootstrap 4.1.1 (https://getbootstrap.com). Interactive data visualization is supported by echarts 4.0 (http://echarts.baidu.com) and jQuery v3.3.1 (https://jquery.com). The web interface is compatible with all web browsers and may work best on Google Chrome, Firefox, or Safari.

### GO enrichment analysis

The R package clusterProfiler v4.2.1 [40] was used to identify enriched ‘Biological Process’ GO terms for the potential disease-causing genes. The parameters were set as follows: pvalueCutoff = 0.01, qvalueCutoff = 0.01, and pAdjustMethod = “BH”. Subsequently, the semantic similarities of the top 20 enriched terms were calculated by GOSemSim v2.20.0 [41] with the following parameter: measure = ‘Wang’.

## Supplementary Material

qzae068_Supplementary_Data

## Data Availability

DRED is freely accessible at http://omicslab.genetics.ac.cn/dred.

## CRediT author statement

**Qingqing Shi:** Data curation, Software, Writing – original draft, Writing – review & editing, Visualization. **Min Dai:** Data curation, Software, Methodology, Writing – original draft, Writing – review & editing, Verification. **Yingke Ma:** Data curation. **Jun Liu:** Data curation. **Xiuying Liu:** Data curation. **Xiu-Jie Wang:** Conceptualization, Methodology, Writing – original draft, Writing – review & editing, Funding acquisition. All authors have read and approved the final manuscript.
