# Supplementary material for: DRED: A Comprehensive Database of Genes Related to Repeat Expansion Diseases
Source: Genomics Proteomics Bioinformatics. 2024 Sep 30;22(5):qzae068. doi: 10.1093/gpbjnl/qzae068 (PMC11696699; doi:10.1093/gpbjnl/qzae068)
Supplement: qzae068_Supplementary_Data [file qzae068_supplementary_data.zip › supplementary material captions.docx]

**Supplementary material**

**Table S1 Summary of the integrated data sources in DRED**

**Table S2 ChIP-seq data used for CTCF-binding peak identification**

**Table S3 Features used for potential disease-causal gene prediction and evaluation**
