# Supplementary material for: DRED: A Comprehensive Database of Genes Related to Repeat Expansion Diseases
Source: Genomics Proteomics Bioinformatics. 2024 Sep 30;22(5):qzae068. doi: 10.1093/gpbjnl/qzae068 (PMC11696699; doi:10.1093/gpbjnl/qzae068)
Supplement: qzae068_Supplementary_Data [file qzae068_supplementary_data.zip › Table S1.docx]

**Table S1 Summary of integrated data sources in DRED**

| **Category** | **Data source** |
| --- | --- |
| Basic information | Ensembl, NCBI, UCSC Genome Browser |
| Disease association | DisGeNET, OMIM |
| Gene function | Gene Ontology, UniProt |
| Gene expression | BioGPS |
| Genetic variant | 1000 Genomes, ClinVar, ESP, ExAC, gnomAD, Kaviar |
| Genomic feature | 3D Genome Browser, ENCODE, UCSC Table Browser |
| Pathway annotation | KEGG, Reactome |
