# Supplementary material for: DRED: A Comprehensive Database of Genes Related to Repeat Expansion Diseases
Source: Genomics Proteomics Bioinformatics. 2024 Sep 30;22(5):qzae068. doi: 10.1093/gpbjnl/qzae068 (PMC11696699; doi:10.1093/gpbjnl/qzae068)
Supplement: qzae068_Supplementary_Data [file qzae068_supplementary_data.zip › Table S2.docx]

**Table S2 ChIP-seq data used for CTCF-binding peak identification**

| **ENCODE accession No.** | **Sample type** |
| --- | --- |
| ENCFF202NVV | Ovary from female adult (51 years old) |
| ENCFF228IAW | H1-hESCs |
| ENCFF362CYM | GM12864 cells |
| ENCFF557KXD | Neural cell originated from H1-hESC |
| ENCFF588VGU | GM12801 cells |
| ENCFF609IPZ | Human osteoblasts |
| ENCFF617JSF | HCT116 cells |
| ENCFF655TAP | Tibial nerve from female adult (51 years old) |
| ENCFF750BRK | Cardiac muscle cells |
| ENCFF788JIP | A549 cells |
