# Supplementary material for: DRED: A Comprehensive Database of Genes Related to Repeat Expansion Diseases
Source: Genomics Proteomics Bioinformatics. 2024 Sep 30;22(5):qzae068. doi: 10.1093/gpbjnl/qzae068 (PMC11696699; doi:10.1093/gpbjnl/qzae068)
Supplement: qzae068_Supplementary_Data [file qzae068_supplementary_data.zip › Table S3.docx]

**Table S3 Features used for potential disease-causal gene prediction and evaluation**

| **Feature** | **Value** | **Weight** |
| --- | --- | --- |
| gnomAD-I^a^ | 0/1^b^ | 0.473 |
| CpG island in proximity region (CpG_P) | 0/1 | 0.409 |
| CpG island overlapping with repeat track (CpG_O) | 0/1 | 0.353 |
| Kaviar-M^a^ | 0/1 | 0.327 |
| Kaviar-I | 0/1 | 0.304 |
| Gene-disease association in DisGeNET (DGN) | 0/1 | 0.286 |
| gnomAD-M | 0/1 | 0.249 |
| CTCF in proximity region (CTCF) | $x/10$^c^ | 0.209 |
| ExAC-I | 0/1 | 0.191 |
| Gene-disease association in OMIM (OMIM) | 0/1 | 0.136 |
| ExAC-M | 0/1 | 0.123 |
| TAD boundaries (TADb) | $x/40$^c^ | 0.121 |
| ESP-M | 0/1 | 0.057 |
| 1000 Genomes-M (1KG_M) | 0/1 | 0.053 |
| ClinVar-M | 0/1 | 0.043 |
| *Alu* element in proximity region (Alu) | 0/1 | 0.042 |
| 1000 Genomes-I (1KG_I) | 0/1 | 0.035 |
| ESP-I | 0/1 | 0.032 |
| ClinVar-I | 0/1 | 0.011 |

*Note*: a, Features with suffix I means insertions of at least one repeat unit in the repeat track, features with suffix M means mutations other than insertions in the repeat track. b, 1 indicates a feature is observed for the repeat track, whereas 0 indicates the feature is not observed. c, $x$ is the number of datasets in which the repeat track has the corresponding feature.
